# Isoliquiritigenin blunts osteoarthritis by inhibition of bone resorption and angiogenesis in subchondral bone

**DOI:** 10.1038/s41598-018-19162-y

**Published:** 2018-01-29

**Authors:** Baochao Ji, Zhendong Zhang, Wentao Guo, Hairong Ma, Boyong Xu, Wenbo Mu, Abdusami Amat, Li Cao

**Affiliations:** 1 Department of Orthopaedics, First Affiliated Hospital of Xinjiang Medical University, 137 South LiYuShan Road, Urumqi, Xinjiang 830054 China; 2 Research Institute of Clinical Medicine, First Affiliated Hospital of Xinjiang Medical University, 137 South LiYuShan Road, Urumqi, Xinjiang 830054 China

## Abstract

Isoliquiritigenin (ISL), a natural flavonoid extracted from licorice, has been demonstrated to exert attenuation of osteoclastogenesis and anti-angiogenesis activity in a wide variety of cells. Here, we first evaluated the effects of ISL on pathogenesis of osteoarthritis in a mouse model of OA. The data showed that ISL blunted progression of OA and lowered the Osteoarthritis Research Society International (OARSI)-Modified Making Score and protected the articular cartilage. The thickness of calcified cartilage zone was significantly decreased in ISL-treated ACLT mice compared with vehicle group. ISL increased expression level of lubricin and decreased collagen X (Col X), matrix metalloproteinase-13 (MMP-13). Moreover, ISL reduced aberrant active subchondral bone remodelling, including lowered trabecular pattern factor (Tb.pf) and increased bone volume/tissue volume (BV/TV, %) and thickness of subchondral bone plate (SBP) compared with vehicle-treated group. The results of immunostaining further revealed that ISL directly reduced RANKL-RANK-TRAF6 singling pathway induced osteoclastogenesis, prevented abnormal bone formation through indirect inhibition of TGF-β release. Additionally, ISL exerts anti-angiogenesis effects in subchondral bone through direct suppression of MMP-2. These results indicated that ISL attenuates progression of OA by inhibition of bone resorption and angiogenesis in subchondral bone, indicating that this may be a potential preventive therapy for OA.

## Introduction

Osteoarthritis (OA) is the most frequent and costly form of arthritis, characterized by slowly, progressive, ultimately degenerative disorder confined to diarthrodial joints. The disease not only causes loss of articular cartilage but also involves the entire joint including inflammation of synovium, formation of osteophyte, sclerosis of subchondral bone^1,2^. These pathologies lead to chronic joint pain, movement limitation and eventually disability. Osteoarthritis to varying degrees happened in an approximate 10–15% of adults over 60 all around the world^3^. The cost of OA was estimated to make up of 0.50% of a country’s gross domestic product^4^. Even so, available agents only provide temporarily symptomatic relief but with numerous side-effects at present, and no medication has been currently approved by the FDA or any other agencies worldwide for OA management, which is mainly due to our limited understanding of the pathogenesis of OA. Therefore, this insufficient area of research is greatly needed as targets for preventive and disease-modifying therapies.

In addition to cartilage degradation, the problem of cartilage defects extending into the underlying subchondral bone has received increasing attention recently^5–8^. Subchondral bone work as a structural girder and shock absorber, which could attenuate about 30% of the loads through joints^9^, and supports superficial articular cartilage^10^. The interaction between the two structures is thought to be a central feature of this process. The subchondral bone consists of the subchondral bone plate and the subarticular spongiosa. It is separated by the cement line from the calcified zone of the articular cartilage^11^. The architecture of subchondral bone is kept by a dynamic balance between modelling and remodelling in response to mechanical stress^12^. Coupled bone remodelling process depend on temporally and spatially regulation of osteoclast and osteoblast activity^11^. Several animal studies have confirmed that high bone remodelling takes place in the early stages, and might trigger the onset of OA^13–15^. Furthermore, recent research indicated that excessive activation of TGF-β1 by elevated osteoclast bone resorption uncouples bone resorption and formation, which contributing to the sclerotic phenotype in the subchondral bone in OA animal models^16^, and the progression of OA could be attenuated by inhibiting TGF-β1 signalling^17^. Additionally, vascularisation in subchondral bone during the progression of OA have also been noted, which couples osteogenesis during bone modeling and remodeling^18^. As a consequence, an agent that could aim at the multiple pathological changes in subchondral bone would be really desired.

**Figure 1:**
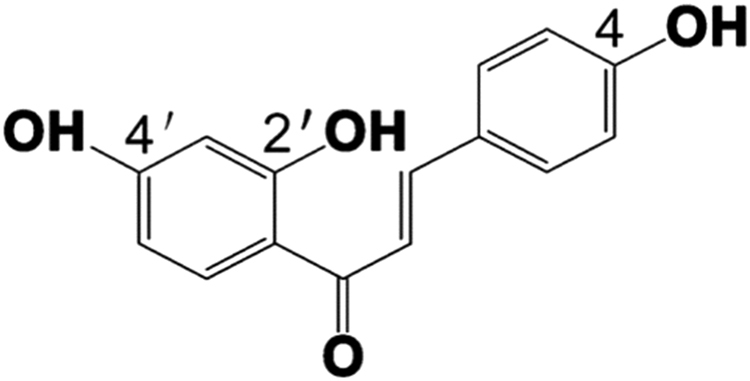
Molecular structure of isoliquiritigenin (ISL, C15H12O4, MW = 256.25).

There has been a recent global trend toward the use of naturally bioactive herbs with anti-inflammatory properties to treat arthritis and other inflammatory diseases^19–21^. Isoliquiritigenin (ISL) (Fig. 1), a natural flavonoid extracted from licorice, has drawn wide attention due to its lots of biologic activities, including anti-diabetic, anti-cancer, anti-oxidant as well as anti-inflammatory properties and its proven pharmacologic safety^22–24^. It is also used in Western countries widely for culinary purpose^25^. Recently, it is reported that ISL could inhibit high glucose (HG)-upregulated connective tissue growth factor (CTGF) and tissue inhibitor of MMP-2 (TIMP-2) expression via disturbing transforming growth factor β1(TGF-β1) signaling in human mesangial cells (HRMC), as evidenced by TGF-β receptor I kinase (TGF-β RI) inhibitor^26^. ISL has also been reported to suppress receptor activator of nuclear factor kappa-B ligand (RANKL)-induced osteoclastogenesis and inflammatory bone loss *in vitro* via RANKL-RANK-TRAF6, mitogen-activated protein kinases (MAPK), IκBα/NF-κB, and AP-1 signaling pathways^27^. Furthermore, ISL has been showed significantly inhibited the RANKL/osteoprotegerin (OPG) ratio by reducing the production of RANKL and restoring OPG production to control levels in hFOB1.19 cells stimulated with conditioned medium (CM) of MDA-MB-231 cells at non-toxicity concentrations^28^. In addition to regulate bone metabolism, it is reported that ISL could induce anti-angiogenic effects, including inhibition of breast cancer neoangiogenesis via suppress vascular endothelial growth factor (VEGF)/VEGFR-2 signaling pathway and matrix metalloproteinase-2 (MMP-2)^29^, suppression platelet-derived growth factor-BB (PDGF-BB) which secreted by preosteoclasts induces angiogenesis during coupling with osteogenesis^30^. Seemingly, ISL could target the multiple pathological changes of OA. However, a search of Medline, PubMed, and Medscape revealed no article on the subject of ISL for treatment of OA. In this situation, we investigated whether ISL has potential effect for preventive treatment of OA, including delaying articular cartilage degeneration and subchondral bone sclerosis in mice anterior cruciate ligament transection (ACLT) models by inhibiting osteoclastogenesis, TGF-β-dependent Smad2/3 phosphorylation and angiogenesis.

## Results

### ISL preserved articular cartilage in ACLT mice

**Figure 2:**
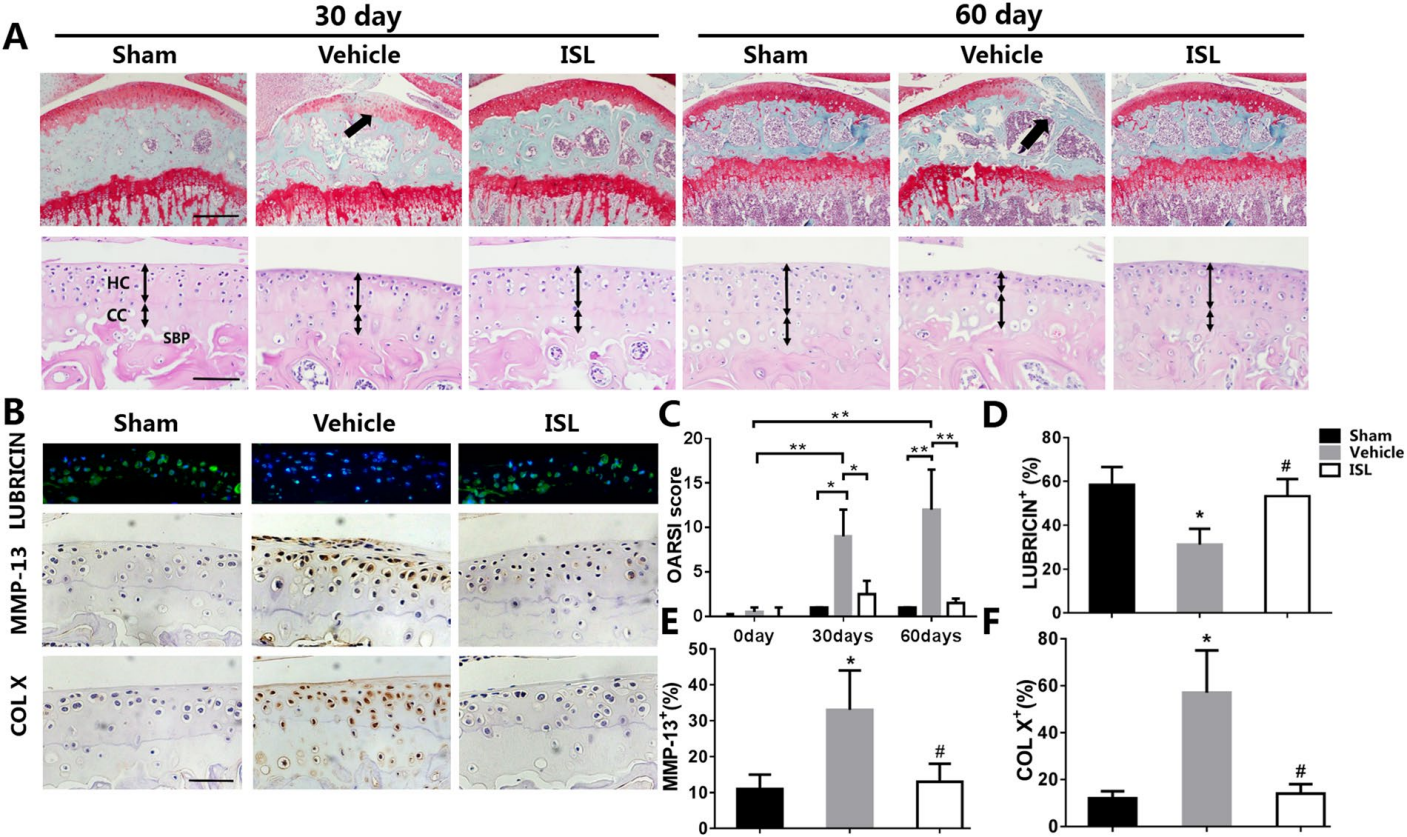
Isoliquiritigenin (ISL) protects articular cartilage after anterior cruciate ligament transection (ACLT) in mice. (**A**) Safranin O and fast green staining (top). Solid arrows indicate proteoglycan loss and cartilage destruction at 30 and 60 days post operation. Scale bar, 200 mm. The thickness of calcified cartilage (CC) and hyaline cartilage (HC) are marked by double-headed arrows in HE staining (bottom). Scale bars, 100 mm. (**B**,**D**,**E**,**F**) The expression of lubricin (**B**-top, **D**), matrix metalloproteinase (MMP) 13 (**B**-middle, **E**) and COL X (**B**-bottom, **F**) in articular cartilage 30 days after ACLT were test by immunostaining and quantitative analysis. Scale bar, 100 mm. (**C**) Osteoarthritis Research Society International (OARSI)–modified Mankin scores of articular cartilage at different time-points after surgery. Sham = incision was made and sutured immediately. Vehicle = ACLT-surgery treated with vehicle. ISL = ACLT-surgery treated with Isoliquiritigenin. n = 8 per group. *p < 0.05 compared with sham or as denoted by bar, **<0.01 compared as denoted by bar; ^#^p < 0.05 compared with the vehicle.

From the Safranin O and fast green staining, significant loss of proteoglycan in vehicle -treated ACLT mice compared with the sham control, which was retention by administrating ISL (40 mg/kg) (Fig. 2A top) at 30 day and 60 day after operation. It is also supported by OARSI scores which were improved in ISL-treated group relative to vehicle group, whereas no difference was noted in ISL versus sham controls (Fig. 2C). Relative to vehicle-treated ACLT mice at 60 day postoperation, decreased thickness of calcified cartilage zone in ISL-treated group was observed from HE staining (Fig. 2A bottom and Table 1). Abnormal expression of MMP-13 and collagen X (Col X) were found in vehicle group compared with the sham control group, which was normalised by administrating ISL as assessed by immunostaining (Fig. 2B,E,F). Conversely, the expression of lubricin (Fig. 2B,D) and Collagen II (COL II) (Supplementary Fig. 3) in vehicle group were significantly decreased, which were improved in ISL-treated group, whereas no difference was noted in ISL versus sham controls.

**Table 1:** Cartilage thickness changes in different group and time-points (10× magnified images; mean ± SD; unit:mm).

Time (days)	HC			CC		
	Sham	Vehicle	Isoliquiritigenin	Sham	Vehicle	Isoliquiritigenin
30	0.78 ± 0.03	0.73 ± 0.04	0.77 ± 0.02	0.37 ± 0.04	0.39 ± 0.04	0.38 ± 0.03
60	0.76 ± 0.19	0.42 ± 0.18	0.72 ± 0.12	0.34 ± 0.16	0.71 ± 0.18	0.41 ± 0.14

The level of significance was set at p < 0.05 and indicated by ‘*’ for the comparison between vehicle-treated group and sham group, or ‘†’ for the comparison between isoliquiritigenin-treated group and vehicle-treated group. CC, calcified cartilage; HC, hyaline cartilage.

### ISL normalized high subchondral bone remodeling in ACLT mice

**Figure 3:**
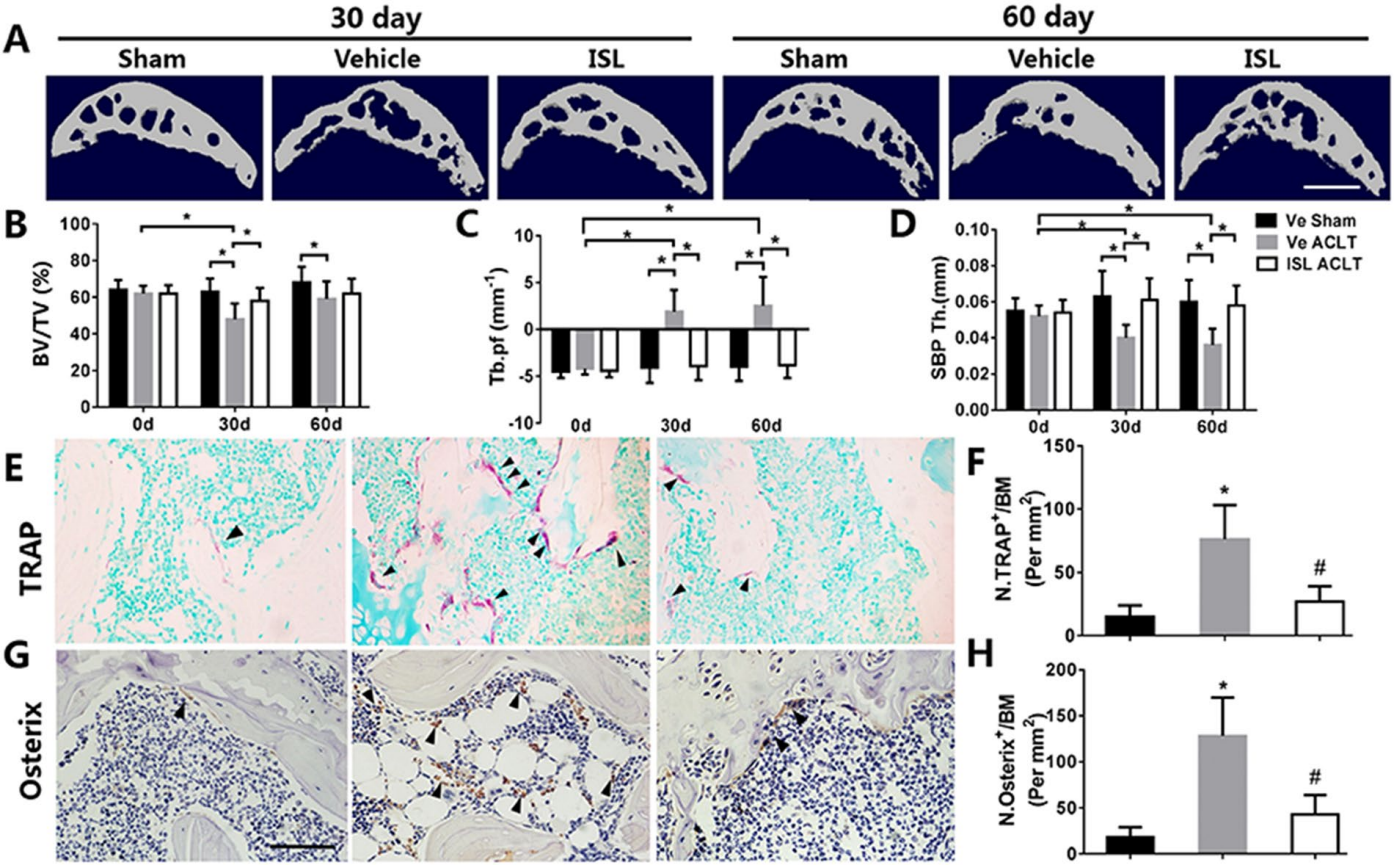
Isoliquiritigenin (ISL) Inhibits aberrant active subchondral bone remodeling after anterior cruciate ligament transection (ACLT) in mice. (**A**) 3D micro-CT image of sagittal views of subchondral bone medial compartment at 30 and 60 days after operation. Scale bar, 500 mm. (**B**–**D**) Quantitative micro-CT analyzes microarchitecture of subchondral bone medial compartment, including bone volume/tissue volume (BV/TV, %)(**B**), trabecular pattern factor (Tb.pf) (**C**) and subchondral bone plate thickness (**D**). Immunostaining and quantitative analysis of TRAP (**E**,**F**) positive cells in tibial subchondral bone at 14 days post operation. Scale bar, 100 mm. The expression of Osterix positive cells in tibial subchondral bone at 30 days after surgery was evaluated by Immunostaining and quantitative analysis (**G**,**H**). Scale bar, 100 mm. n = 8 per group. *p < 0.05 compared with sham or as denoted by bar; ^#^p < 0.05 compared with the vehicle.

High-resolution Micro-CT was used to access whether the protective role of ISL on the articular cartilage is associated with its potential effect on the microarchitecture of tibial subchondral bone. The results showed that in the vehicle group, the value of bone volume/tissue volume (BV/TV, %) reduced post ACLT, which was abrogated by receiving ISL. Additionally, ISL significantly reduced trabecular pattern factor (Tb.pf) (a parameter of bone resorption) and increased SBP thickness (a parameter of bone formation) post ACLT compared with vehicle treatment and there was no statistically significant difference in these parameters compared with sham controls (Fig. 3A–D). Correspondingly, ISL significantly reduced the number of TRAP-positive osteoclasts and osterix-positive osteoprogenitors postoperation relative to vehicle treatment (Fig. 3E–H). It is also noted that the majority of osterix-positive cells were found in subchondral bone marrow in the vehicle group and relocated to the bone surface in the ISL-treated mice. The results of these data demonstrated that ISL could normalize aberrant subchondral bone remodeling in ACLT mice.

### ISL inhibits osteoclastogenesis by suppression RANKL-RANK-TRAF6 singling pathway in the subchondral bone of mice

**Figure 4:**
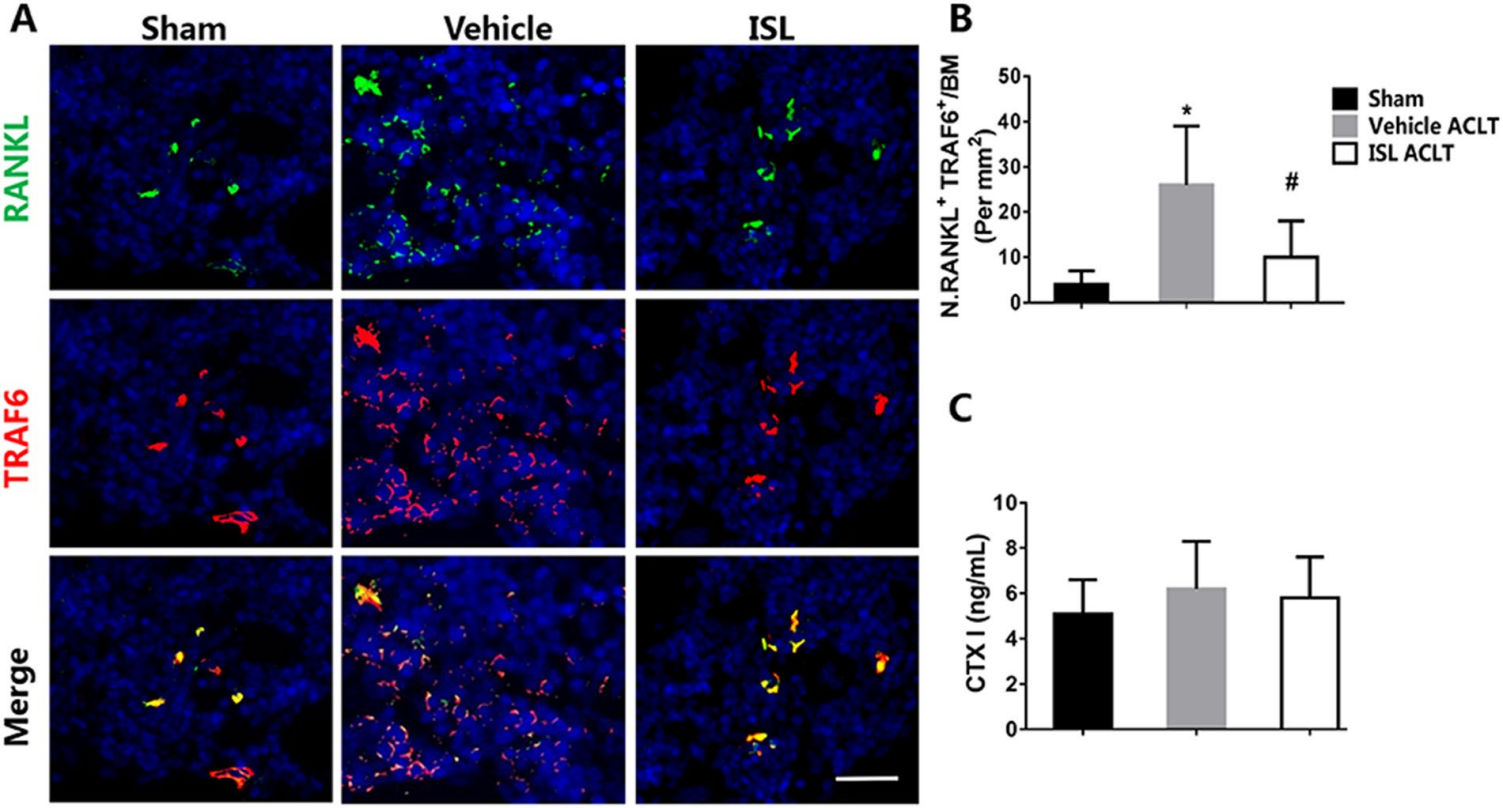
Isoliquiritigenin (ISL) inhibits RANKL-RANK-TRAF6 singling pathway in the subchondral bone of mice after anterior cruciate ligament transection (ACLT). (**A**,**B**) Representative immunofluorescence double staining and quantitative analysis for RANKL (green), TRAF6 (red) and merged images (colocalisation = yellow) 14 days post-operation. Scale bar, 50 mm. (**C**) EIA analysis of C-terminal telopeptide of type I collagen (CTX-I) in peripheral blood plasma 14 days post-surgery. n = 8 per group. *p < 0.05, compared with the sham; ^#^p < 0.05 compared with the vehicle.

Representative immunofluorescence double staining and enzyme immunoassay (EIA) were performed to investigate the potential mechanism of how ISL affect the microstructure of subchondral bone. RANKL-RANK-TRAF6 singling pathway plays crucial roles in osteoclast differentiation and activation. Immunofluorescence double staining showed a significant increase of RANKL and TRAF6 in the subchondral bone marrow of vehicle-treated mice as early as 2 weeks postoperation, whereas ISL-treated mice had equivalent expression compared with sham controls (Fig. 4A,B). However, no difference in the expression level of CTX I in peripheral blood was observed regardless of vehicle or ISL-treated mice 14 days after ACLT operation compare to sham group by using EIA kit (Fig. 4C). The results indicated ISL inhibits osteoclastogenesis by suppression RANKL-RANK-TRAF6 singling pathway in the subchondral bone.

### ISL inhibits TGF-β release in subchondral bone medial compartment by suppression osteoclastogenesis

**Figure 5:**
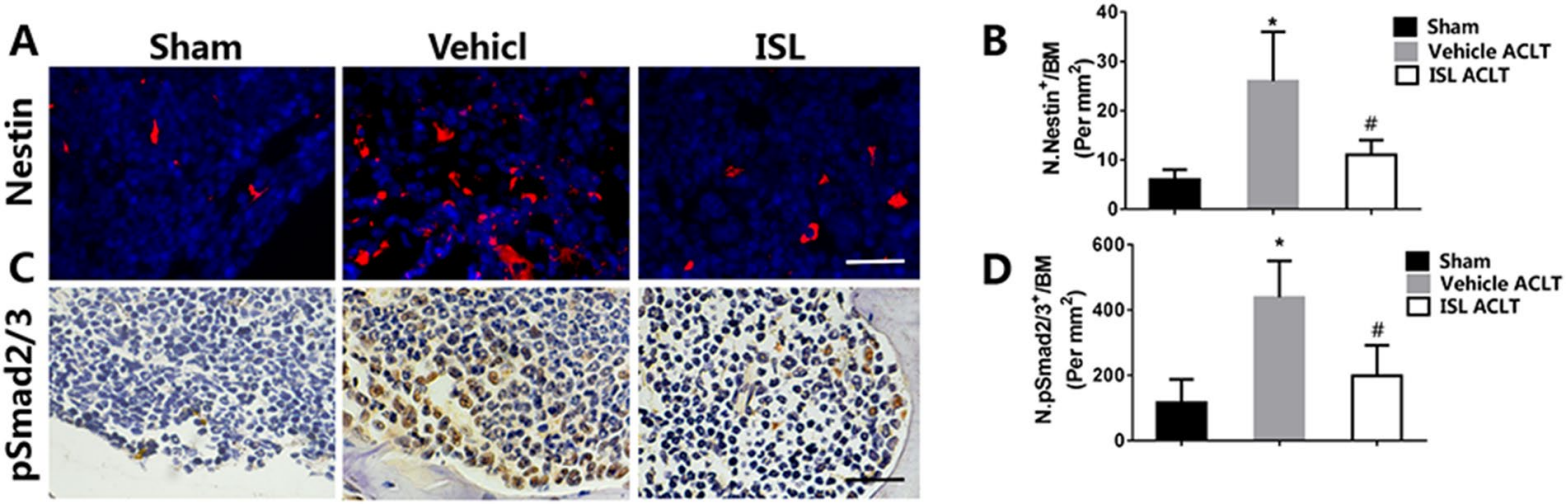
Isoliquiritigenin (ISL) inhibits expression of nestin^+^MSC in subchondral bone medial compartment by suppression TGF-β1/pSmad2/3 singling pathway after anterior cruciate ligament transection (ACLT). (**A**,**C**) Immunostaining and (**B**,**D**) Quantitative analysis for nestin^+^ and pSmad2/3-positive cells in sagittal sections of subchondral bone medial compartment 30 days post-surgery. Scale bar, 100 mm. n = 8 per group. *p < 0.05 compared with the sham and ^#^p < 0.05 compared with the vehicle.

**Figure 6:**
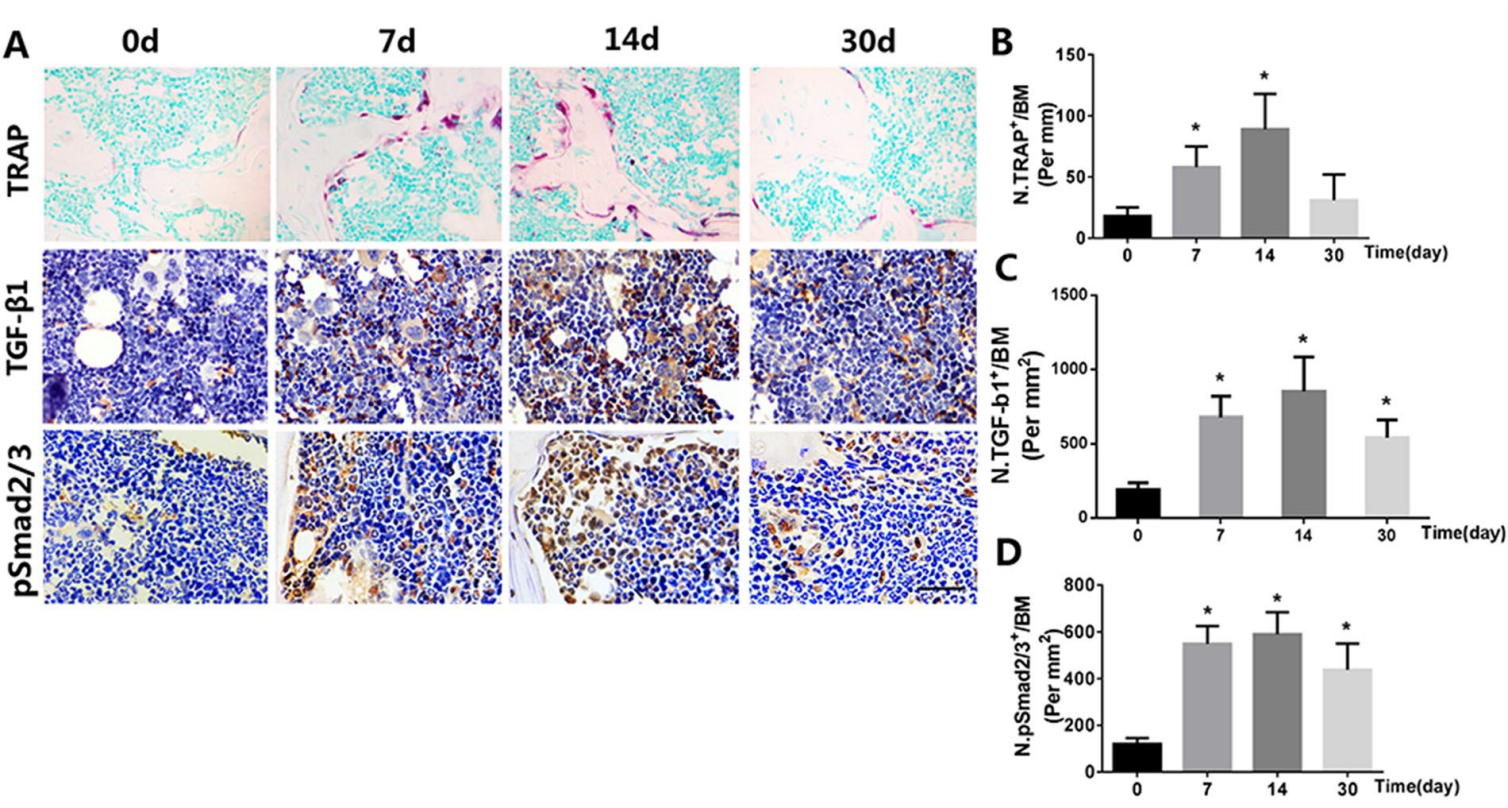
Isoliquiritigenin (ISL) inhibits TGF-β release in subchondral bone medial compartment by suppression osteoclastogenesis after anterior cruciate ligament transection (ACLT). Immunostaining and quantitative analysis for TRAP (**A**-top, **B**),TGF-β1 (**A**-middle, **C**) and pSmad2/3-positive cells (**A**-bottom, **D**) in sagittal sections of subchondral bone medial compartment at 0, 7, 14 and 30 days after surgery. Scale bar, 100 mm. n = 8 per group. *p < 0.05 compared with the sham and ^#^p < 0.05 compared with the vehicle.

Immunofluorescence staining of nestin showed that ISL significantly decreased the number of mesenchymal/stromal stem cells (MSCs) in the subchondral bone after ACLT operation compared with vehicle group, and no statistical difference was found between the ISL-treated and sham group (Fig. 5A,B). As High concentrations of TGF-β1 induced formation of nestin-positive MSC clusters, leading to formation of marrow osteoid islets accompanied by high levels of angiogenesis^17^, we therefore investigated whether ISL could inhibits TGF-β activity in MSCs. Immunohistochemistry staining indicated that pSmad2/3-positive cells in the subchondral bone of vehicle-treated group were significantly increased and reversed to similar levels comparable with sham control by administering ISL (Fig. 5C,D). With the same developments, TRAP positive osteoclast cells, TGF-β1 and pSmad2/3-positive cells both increased in subchondral bone as early as 7d after ACLT. Then the continued osteoclastic bone resorption results in TGF-β1 and pSmad2/3-positive cells remained at high concentrations until 30d (Fig. 6). These results suggest that ISL could inhibit TGF-β activity in bone marrow MSCs by suppression osteoclastogenesis.

### ISL prevents aberrant microvascular formation in subchondral bone

**Figure 7:**
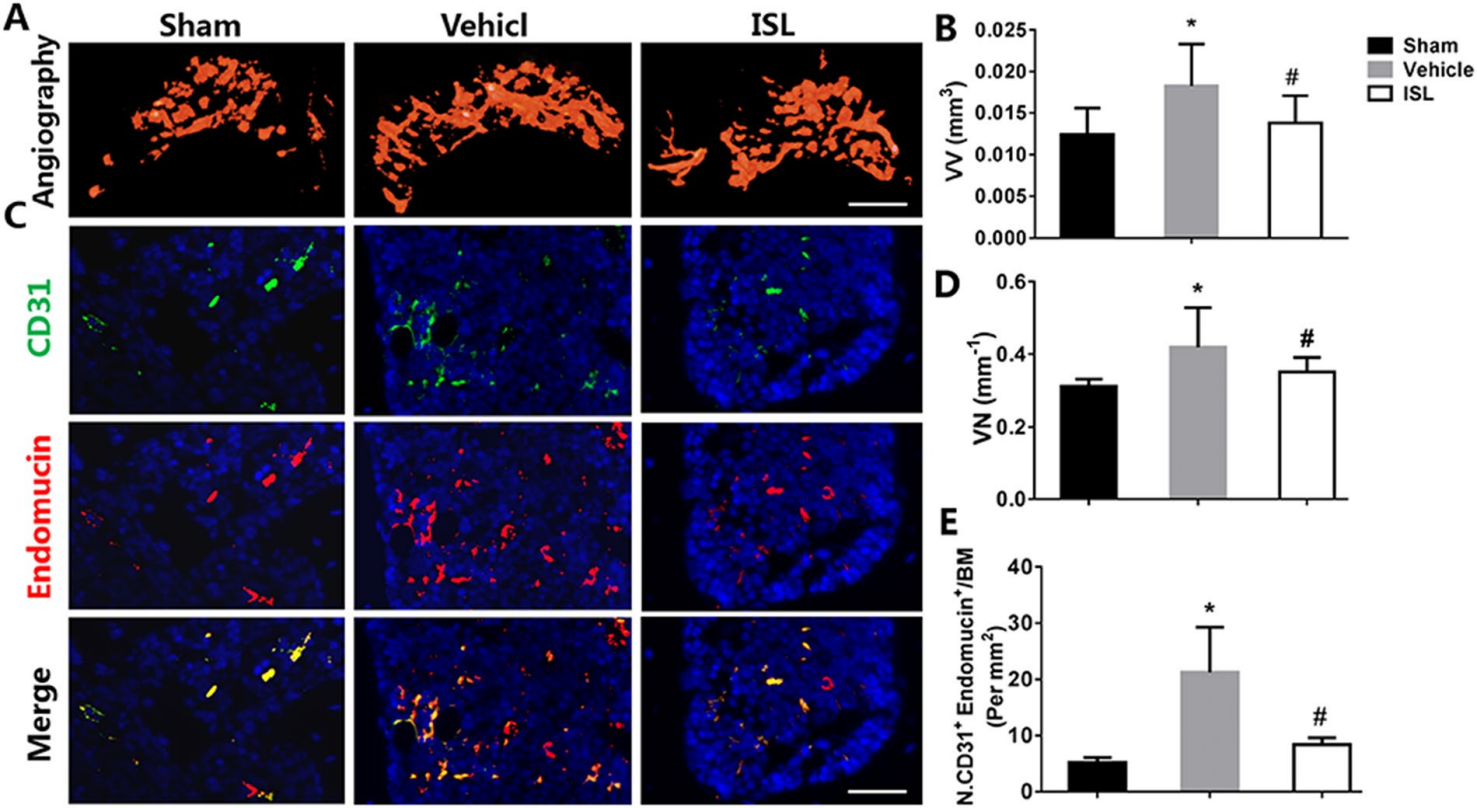
Isoliquiritigenin (ISL) prevents aberrant microvascular formation in subchondral bone of anterior cruciate ligament transection (ACLT) mice. (**A**,**B**,**D**) Micro-CT based microangiography of medial tibial subchondral bone (**A**) 30 days post-operation, with a quantification of vessel volume (**B**) and vessel number (VN) (**D**). Scale bar, 500 mm. (**C**,**E**) Immunofluorescence double staining (**C**) and quantification (**E**) of CD31 (green), endomucin (red) and merged images (colocalisation = yellow) 1 month after surgery. Scale bar, 50 mm. n = 8 per group. *p < 0.05 compared with sham and ^#^p < 0.05 compared with vehicle.

**Figure 8:**
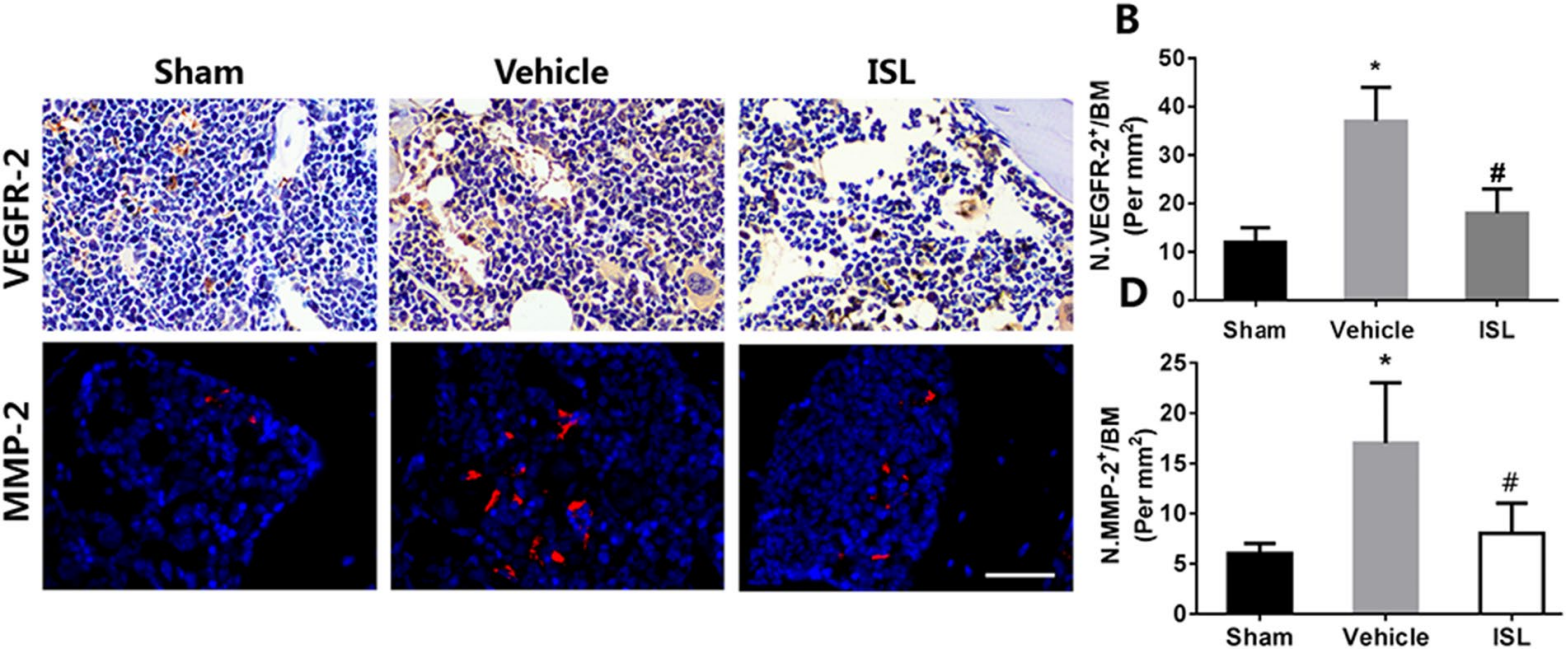
Isoliquiritigenin (ISL) attenuates excessive expression of vascular endothelial growth factor receptor-2 (VEGFR2) and matrix metalloproteinase 2 (MMP-2) in subchondral bone of anterior cruciate ligament transection (ACLT) mice. Immunostaining (**A**) and Quantitative analysis (**B**,**C**) for VEGFR2 and MMP-2 in sagittal sections of subchondral bone medial compartment 30 days post-surgery. Scale bar, 50 mm. n = 8 per group. *p < 0.05 compared with sham and ^#^p < 0.05 compared with vehicle.

At last, Micro CT-based microangiography was used to evaluate the potential effects of ISL on subchondral bone angiogenesis. After ACLT operation 30days, the significantly increased the number of microvascular were observed in the subchondral bone of ACLT mice in vehicle group as well as the volume of microvascular, whereas the aberrant blood vessel formation was prevented by ISL, which retained vessel number and volume similar to sham controls (Fig. 7A,B,D). Furthermore, immunofluorescence double staining for CD31 and endomucin was performed to investigate the potential mechanism of how ISL affect the subchondral bone angiogenesis. Consistently, a significant increase of CD31 and endomucin in the subchondral bone marrow of vehicle-treated mice was found, whereas ISL-treated mice had equivalent expression compared with sham controls (Fig. 7C,E). Immunohistochemical staining of VEGR2 showed that ISL significantly decreased the expression of VEGR2 in the subchondral bone after ACLT operation compared with vehicle group, and no statistical difference was found between the ISL-treated and sham group (Fig. 8A,B). What’s more, the expression level of MMP-2 were also statistically increased in vehicle group. ISL reduced the expression in subchondral bone to similar level compared with sham controls (Fig. 8C,D). The data above indicated that ISL could prevent aberrant blood vessel formation in subchondral bone.

## Discussion

Recent decade, the bioactive small molecules from natural herbage that would treat OA especially with minimal side-effects have been diligently seek^19–21,31,32^. ISL, as familiar small molecules isolated from licorice which is one of the most widely used herbs in traditional oriental medicine, has been used to treat many kinds of ailments ranging from skin diseases to rheumatoid arthritis^31,33^. Our findings broaden the potential application of ISL. In this study, we have found that ISL blunted mechanically induced OA in mice by inhibition of RANKL-RANK-TRAF6-induced osteoclastogenesis, excessive TGF-β release and angiogenesis in subchondral bone. It has been suggested that subchondral bone participates in the early stage of OA. To test whether the level of bone remodelling might modify the metabolism of cartilage, we used an ACLT mice model, which has been widely described and shown the appropriate histological and biochemical changes associated with OA progression^34,35^. As a result of instability of mechanical loading on such joints, for example, excessive body weight and weakening muscles during aging, the subchondral bone and calcified cartilage zone undergo changes^36^. Only 7 days after operation, the number of TRAP-positive osteoclast cells in the subchondral bone has significantly increase in vehicle-treated group, then the continued osteoclastic bone resorption remained at high concentrations until 30 days (Fig. 6). The results of Micro-CT therefore showed aberrant active subchondral bone remodeling in ACLT mice 30 days postoperation (Fig. 3A–D). However, although a significant loss of proteoglycan in articular cartilage in vehicle group were also observed at same time point (30 days postoperation), thickness of calcified cartilage zone had no obvious change until 60 days after ACLT operation (Fig. 2A). These data further strongly supported the theory that there is a cross-talk between subchondral bone and cartilage, an aberrant active bone remodeling may occur prior to final cartilage degeneration. Maintaining the microstructural integrity of subchondral bone provides an essential physiological environment for articular cartilage.

**Figure 9:**
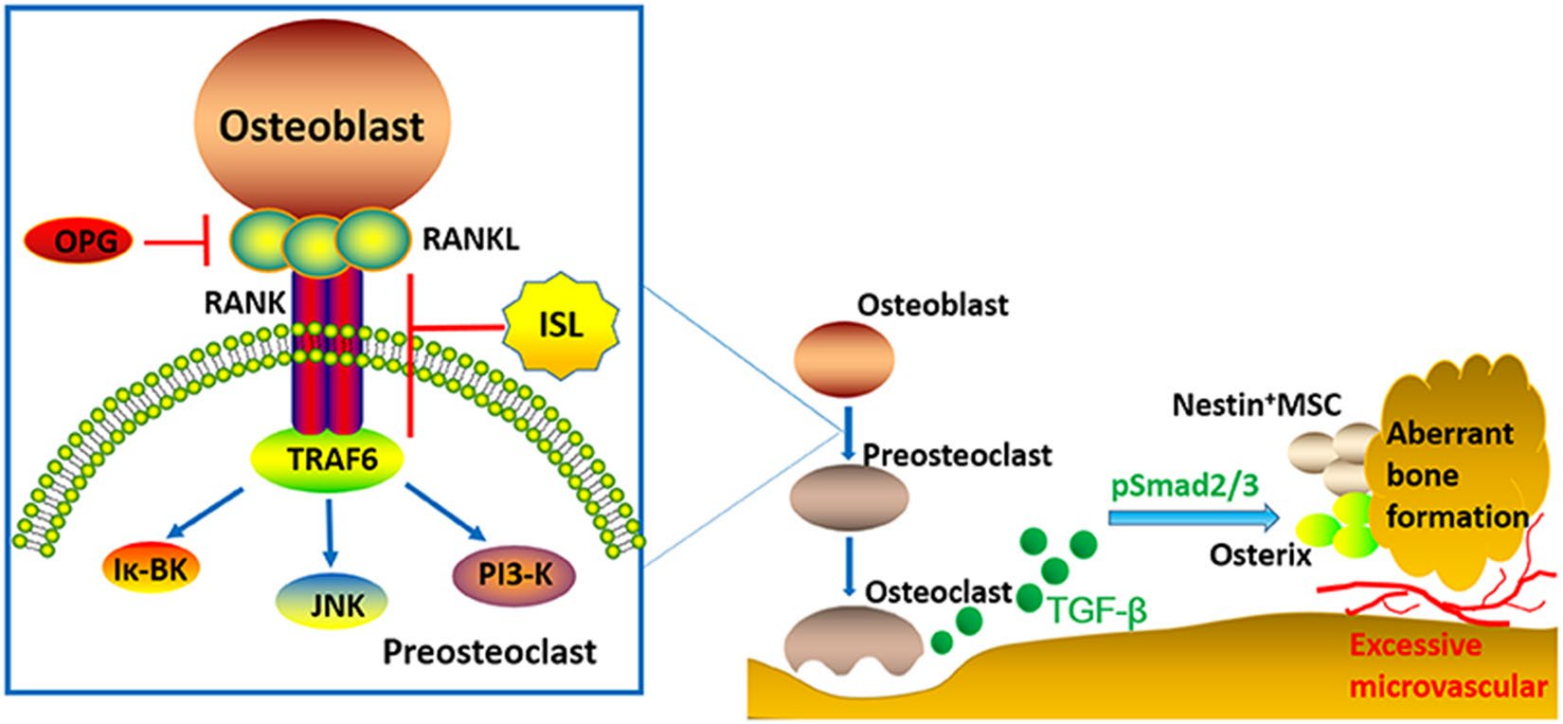
Molecular mechanism of Isoliquiritigenin (ISL) on inhibiting progression of osteoarthritis. The differentiation of osteoclast involves osteoblast and preosteoclast. RANKL is expressed on the surface of osteoblasts, which bind its receptor RANK on the surface of preosteoclast to induce the trimerization and activation of signaling-adaptor molecule tumor necrosis factor receptor-associated factor 6 (TRAF6). It subsequently activates a cascade of events that lead to the differentiation of osteoclast. The abnormal mechanical loading increases subchondral bone resorption to release more active TGF-β which stimulate increases in the number of MSCs and osteoprogenitors in the bone marrow, which lead to aberrant bone formation and angiogenesis for osteoarthritis progression. ISL inhibits RANKL-RANK-TRAF6 singling pathway in preosteoclast to reduce release of TGF-β in the subchondral bone and therefore prevent a cascade of events that lead to the development of osteoarthritis.

Osteoclasts, bone-specialized multinucleated cells, are derived from hemopoietic progenitors of the macrophage lineage through a differentiation process which primarily mediated by two key cytokines, namely macrophage colony-stimulating factor (M-CSF) and receptor activator of nuclear factor-κB ligand (RANKL)^37^. M-CSF is important for the proliferation and survival of osteoclast precursors^38^. RANKL as a member of the tumor necrosis factor family is expressed on the surface of osteoblasts and stromal cells, and plays crucial roles in osteoclast differentiation and activation^37^. The binding of RANKL with its receptor RANK induces the trimerization and activation of signaling-adaptor molecule tumor necrosis factor receptor – associated factor 6 (TRAF6) which as initial cytokines subsequently leads to the activation of the nuclear factor-κB (NF-κB) pathway as well as three well-known mitogen-activated protein kinase (MAPK) pathways, including p38 MAPK, extracellular signal-regulated kinase (ERK) 1/2, and c-Jun-N-terminal kinase (JNK)^37^ (Fig. 9). With increasing number of research pay close attention to the role of subchondral bone in the pathomechanism of OA, osteoclasts recently is considered as a potential target in the treatment^39,40^. Alendronate was used as osteoclast-blocking agents, prevented OA progression in rats by blocking bone resorption^41^. Additionally, Pamidronate was also administered in mice with overexpressing Runx2 which received an partial medial meniscectomy to induce OA. Six weeks after surgery, pamidronate prevents the increase in the OA score in Runx2-Tg mice by inhibition bone resorption^42^. Furthermore, Risedronate reduced the levels of collagen type II (CTX-II), a cartilage degradation marker, in a dose-dependent matter^43^ and patients with both low collagen type II and collagen type I levels had the lowest risk of OA progression^44^. However, even with so much research, the potential mechanism of osteoclast-blocking agents in the treatment of OA have not been elucidated at the molecular level.

Inspiringly, a recent study reported that altered mechanical loading could increase subchondral bone resorption which then release more active TGF-β in ACLT OA mice. These abnormal TGF-β signalling would interrupt coupled bone remodelling, recruiting.

MSCs to form aberrant osteoid islets in bone marrow as opposed to bone resorption pits for coupled bone resorption. It changed microstructure of subchondral bone and ultimately lead to articular cartilage degeneration^17^. Based on this, the current study demonstrated that ISL directly inhibited osteoclastogenesis by suppression RANKL-RANK-TRAF6 singling pathway in the subchondral bone of mice and indirectly suppressed TGF-β release from bone matrix, then prevented aberrant migration of MSCs, re-established coupled bone remodelling and protected articular cartilage. In detail, osterix-positive osteoprogenitors from MSCs, are precursor cells of osteoblast. During the normal remodeling process, osteoblasts and their progenitors are located primarily at the resorption site on the bone surface. However, the abnormal mechanical loading leads to the excessive release of TGF-β which due to increased bone absorption. These excessive TGF-β mediate further differentiation of MSCs into osteoblast precursors and eventually lead to commitment of osteoprogenitors *in-situ* in bone marrow cavities. These clustered bone marrow osteoprogenitors may lead to osteoid islets in the subchondral bone marrow which are visualized as bone marrow lesions during MRI, which have been identified as a prognostic factor of OA progression^45^. However, ISL could inhibit excessive release of TGF-β in bone marrow by suppression osteoclastogenesis through targeting RANKL-RANK-TRAF6 singling pathway in preosteoclast and therefore prevent a cascade of events that lead to the development of osteoarthritis (Fig. 9). Nevertheless, although ISL has been reported could disturb TGF-β1 signaling in human mesangial cells (HRMC)^26^, whether ISL could directly inhibit phosphorylation of Smad2 (pSmad2) in MSCs in ACLT mice has not yet been researched in this study, which are required in future studies. Besides, we found no difference in the expression level of CTX I in peripheral blood regardless of vehicle or ISL-treated mice 14 days after ACLT operation compare to sham group (Fig. 4C). A speculation is performed that unlike osteoporosis, topical bone resorption in ACLT OA mice may not enough to cause change in the value of CTX I in peripheral blood.

Angiogenesis is critical for bone health to transport nutrients, oxygen, minerals and metabolic wastes^46^. This process is coupled with bone formation in regulating bone remodeling. However, aberrant microvascular information in subchondral bone is a known pathological feature of OA^47^. From Micro-CT analysis, ISL reduces the number and volume of microvascular in the subchondral bone of ACLT mice to the similar level of sham group. At the molecular level, a significant increase of CD31 and endomucin in the subchondral bone marrow of vehicle-treated mice was observed, whereas ISL-treated mice had equivalent expression compared with sham controls. In order to investigate the potential mechanism, we further performed an immunohistochemistry assay on VEGFR2 and MMP-2. VEGF has been identified as the most important pro-angiogenic factor^48,49^. After binding with VEGF receptors on the surface of endothelial cell, which sequentially promote endothelial cells recruitment and proliferation^50,51^. MMP-2 is one kind of membrane-associated neutral endopeptidases which produced by endothelial cells. It can promote angiogenesis by regulation of cell-extracellular matrix interactions. ISL normalized high expression of VEGFR2 and MMP-2 in vehicle-treated ACLT mice. Besides that, TGF-β signalling in endothelial progenitor cells can also induce angiogenesis^52^. A recent study shown that TGF-β inhibition can reduce angiogenesis in subchondral bone in ACLT OA mice^17^. Therefore, the results of this research indicated that ISL prevents aberrant blood vessel formation in subchondral bone by direct suppression of MMP-2, indirect inhibition of TGF-β signalling. Moreover, platelet derived growth factor-BB (PDGF-BB) was secreted by preosteoclasts and reported could prepares angiogenesis for anticipating bone formation in addition to recruitment of MSCs^53^. Although ISL has been reported could attenuate adipose tissue fibrosis by suppression the expression level of PDGF-BB^30^, the effect of ISL on this growth factor in subchondral bone of ACLT mice has not yet been researched in current study, which may be another additional unexplored mechanisms.

ISL is extracted from licorice with extensive sources. This perennial herb is mainly distributed in southern Europe and Asia. ISL, with proven extractive technique, cheap price, and reliable safety, is not only applied in medical field, but also used as food additive for many years. Unlike osteoclast-blocking agents or TGF-β inhibitor, the small molecule ISL has lots of biologic activities. The data of current study further demonstrated that there is a cross-talk between subchondral bone and cartilage, an aberrant active bone remodeling may occur prior to final cartilage degeneration. ISL blunted OA progression and protected articular cartilage in mice ACLT models by two approaches. On the one hand, it inhibited aberrant subchondral bone remodeling in early OA, including directly reduced RANKL-RANK-TRAF6 singling pathway induced osteoclastogenesis, prevented abnormal bone formation through indirect inhibition of TGF-β release. On the other hand, ISL abrogated aberrant microvascular formation in subchondral bone by direct suppression of MMP-2, indirect inhibition of TGF-β signalling. These results suggest that ISL target subchondral bone in early OA can be fairly effective in ACLT mice, indicating that this may be a potential preventive therapy for OA.

## Materials and Methods

### Animals

#### Ethical statement

All procedures complied with the guidelines of the Association for Assessment and Accreditation of Laboratory Animal Care, and the protocol was approved by the Institutional Animal Care and Use Committee of First Affiliated Hospital of Xinjiang Medical University (protocol number IACUC20160616-08).

### Mice

C57BL/6 J is one kind of inbred mouse with characteristic of good comparability and consistent stress, which is commonly used by tumor, physiology, immunology and genetics research. What is more, it is the most commonly used rodents in OA study. The maturity of C57BL/6 J mice were 10 weeks, and in order to rule out the effect of estrogen on OA progression, C57BL/6 male mice of 3 months old were purchased from Vital River. Animal feeding environment: temperature 22–25 °C, light - dark cycle 12 hours, relative humidity 60%, all mice free to drink water. The weight of all mice were between 25 and 29 gram before modeling. All of the mice with free activities and good mental appetite were divided into different groups and underwent different treatment in accordance with the random number table. Preliminary experiment was performed firstly, the optimal dose (40 mg/kg) was identified by dividing the mice into sham group, vehicle-treated and multiple concentrations of ISL-treated group randomly (10, 20 and 40 mg/kg; n = 10 per group). Anterior cruciate ligament of the right knee were transected to generate a destabilized OA animal model. Sham operation was done by opening the joint capsule and then suturing the incision in the right knee of independent mice. Beginning the second day after ACLT surgery, ISL (13766 Sigma, USA) or equivalent volume of vehicle (10% Tween-80) was injected intraperitoneally every other day for 60 days. Mice were sacrificed at 60 d after operation. From Safranin O and fast green staining, one-way analysis of variance (ANOVA) on OARSI score indicated that lower concentration (10 or 20 mg/kg) had minimal effects on chondroprotection (Supplementary Fig. 1B,C). We did not continue to increase the dose because nearly half of mice died in higher concentration (80 mg/kg group) during 2 month post-operation. Beyond cartilage, we evaluated the systemic and local responses in each dose of mice by rank-sum test, and no statistical differences were found between the groups with lower concentration (10, 20 and 40 mg/kg) (p > 0.05). But the mice which received 80 mg/kg have worse systemic responses than other groups (p > 0.05) (Supplementary Fig. 1D). Furthermore, the weight and organ coefficient of mice with different dose were compared by One-way ANOVA. The results showed that no statistical differences were found between the groups with lower concentration (10, 20 and 40 mg/kg) (p > 0.05). However, the value of the survival mice which received 80 mg/kg were decreased significantly (p < 0.05) (Supplementary Fig. 1E). Beyond that, taking into account ISL is one kind of phytoestrogen, negative effects may be observed at higher doses. We have compared the ISL and the vehicle group in the non-ACLT mice (sham + ISL). The non-ACLT mice were randomized into sham group, vehicle group, and multiple dose of ISL-treated group (10, 20, 40 and 80 mg/kg; n = 10 per group). Beginning the second day after sham surgery, ISL or equivalent volume of vehicle (10% Tween-80) was injected intraperitoneally every other day for 60 days. Mice were sacrificed at 60d after operation. From HE staining, no statistical differences in thickness of hyaline cartilage were found between the groups with lower concentration (10, 20 and 40 mg/kg) (p > 0.05). Nevertheless, the indicator of the survival mice which received 80 mg/kg were decreased significantly (p < 0.05) (Supplementary Fig. 2). Therefore, in formal experiment, the mice were randomized to sham group, ACLT + vehicle group, and ACLT + ISL (40 mg/kg) group (n = 48 per group). Follow the protocol above, 8 mice were separately sacrificed at 0,7,14, 30 and 60 d post operation; with another 8 in each group specifically for angiography were sacrificed at 30d post operation.

### Micro-CT analysis

Immediately after euthanization, entire knee joints without soft tissue were dissected and fixed in 10% buffered formalin overnight. After that, specimens were scanned using high-resolution micro-CT (SkyScan 1176) with the voltage of 50kVp, filter of 0.5 mm AI and resolution of 9 μm per pixel. The data was then reconstructed (NRecon v1.6), analyzed (CTAn, v1.9) and build for 3D model visualization (CTVol, v2.0). The sagittal view of the entire medial compartment of the tibial subchondral bone was selected for 3D histomorphometric analysis. The region of interest was defined to cover the whole subchondral bone medial compartment. The following parameters were measured: (1) Bone volume/tissue volume (BV/TV, %), (2) trabecular pattern factor (Tb.pf, mm^−1^) as a parameter of bone resorption, (3) subchondral bone plate thickness (SBP Th, mm) as a parameter of bone formation.

### Micro CT-based microangiography

After the mice were euthanized, right atrium was broken with microscissors. Then the blood circulation system was lavaged with 0.9% warm normal saline solution containing heparin sodium (100U ml^−1^) by a needle inserted into the left ventricle. Thereafter, we pressure 10% neutral buffered formalin to fixed the specimen and washed with heparinized saline solution. In the same channel, radiocontrast agent (Microfil MV-122, Flow Tech) was then injected and the specimens were stored at 4 °C overnight. The knee joint of the mice was harvested and soaked in 10% neutral buffered formalin for 5 days. After that, decalcification was performed with a formic acid-based solution (Cal-Ex II) for 72 h to minimize the influence of surrounding tissues. The specimens were also scanned using high-resolution micro-CT (SkyScan 1176) with the resolution of 9 μm isotropic voxel size.

### Histochemistry, immunohistochemistry and histomorphometry analysis

By the time the mice were euthanized, the right knee joints of mice were dissected, fixed in 10% buffered formalin for 48 h, and decalcified in 10% EDTA (pH 7.4) for 3 weeks. Specimens were embedded in paraffin. Longitudinal-oriented sections of the medial compartment of the joint were cut with 4 μm thick and processed for H&E and Safranin O and fast green staining. The thickness of the hyaline cartilage and calcified cartilage were measured with HE staining (thickness of hyaline cartilage: the distance from the tidemark to articular cartilage surface; thickness of calcified cartilage: the distance from the tidemark to subchondral bone plate (SBP)). In order to provide enough statistical power for detecting the mean differences between groups, 8 mice in each group and eight sequential sections per mouse were measured.

Standard protocol of immunohistochemistry were perform in current study. Sagittal sections of knee joint medial compartment were incubated with primary antibodies against Matrix Metallo Preteinases 13 (MMP-13) (Abcam, 1:100, ab39012), Collagen X (Abcam, 1:100, ab58632), Collagen II (Abcam, 1:400, ab ab34712), phosphorylated Smad2/3 (pSmad2/3) (Santa Cruz Biotechnology Inc., 1:40),TGF-β1(Abcam, 1:50, ab92486), Vascular Endothelial Growth Factor Receptor-2 (VEGFR2) (Abcam, 1:100, ab2349) and osterix (Abcam, 1:500, 22552) overnight at 4 °C. After used a horseradish peroxidase–streptavidin detection system (ZSGB BIO) to detect the immunoactivity, counterstaining was performed with hematoxylin (ZSGB BIO). Tartrate-resistent acid phosphatase (TRAP) staining was performed following a standard protocol (Sigma-Aldrich).

For immunofluorescence staining, the sagittal sections were incubated with primary antibodies against lubricin (Abcam, 1:200, ab28484), RANKL (Abcam, 1:100, ab45039), TRAF6 (Abcam, 1:50, ab40675), nestin (Abcam, 1:100, ab11306), CD31 (Abcam, 1:25, ab28364), endomucin (Santa Cruz, V.7C7, 1:50, sc65495) overnight at 4 °C. Then second antibodies conjugated with fluorescence were incubated for 1 h at room temperature (RT) while avoiding light. Histomorphometric measurement was performed on the entire tibial subchondral bone (Olympus DP26) and quantitative analysis was conducted in a blinded way with cellSens software (Olympus, Int.) The positively stained cells in the subchondral bone or entire articular cartilage were counted with three views per section and eight sequential sections per mouse in each group were accessed (n = 8 per group). All counting was performed blindly by an author who was not one of the experimenters. All stains were repeated four times independently (two sections in each time). The Osteoarthritis Research Society International-modified Manking score was accessed as described by Pritzker *et al*.^54^.

### Serological marker analysis

Serum samples were collected at the time of euthanization and stored at −80 °C. Serum C-terminal telopeptide of type I collagen (CTX-I) levels, a biomarker of bone resorption, were measured using RatLaps (CTX-1) EIA kit (Immunodiagnostic Systems Inc., Gaithersburg, MD, USA). the assays were performed follow the manufacturer’s protocol and all the samples were accessed in duplicate. The absolute concentrations were extrapolated from the standard curves which generated from the kit.

### Statistical analysis

Data are expressed as means ± standard deviation (SD) or medium (25th and 75th percentiles). One-way analysis of variance (ANOVA) were performed for multifactorial comparisons in this study. Kolmogorov-Smirnov test was applied to test the normality of distribution. Data that were not normally distributed were log transformed. Homogeneity of variance was tested by Bartlett test first and then the differences between groups were assessed by *post hoc* multiple comparisons. The Bonferroni test or Dunnett test was used to assess the differences between groups for data without heterogeneity. However, if heterogeneity did exist, the Welch test was used to test the equality of means and the Dunnett’s T3 was used to assess the differences between groups. Dunnett test was used to evaluate the differences of expression of TRAP, TGF-β and pSmad2/3 at 0, 7, 14 and 30 days after surgery. Additionally, this approach was also chosen to assess the differences of OARSI score, Tb.pf and SBP th at 30 and 60 days post-operation between vehicle-treated group and sham group. Statistical tests were carried out using SPSS version 18.0 software (SPSS Inc., Chicago, Illinois). The investigators were blinded to allocation during experiments and outcome assessment. The level of significance was set at P < 0.05 and indicated by “*” for the comparison between vehicle-treated group and sham group, or “#” for the comparison between ISL-treated group and vehicle-treated group.

### Data availability statement

The datasets generated during and/or analysed during the current study are available from the corresponding author on reasonable request.

## Electronic supplementary material

Supplementary information
