# Supplementary material for: Isoliquiritigenin blunts osteoarthritis by inhibition of bone resorption and angiogenesis in subchondral bone
Source: Sci Rep. 2018 Jan 29;8:1721. doi: 10.1038/s41598-018-19162-y (PMC5788865; doi:10.1038/s41598-018-19162-y)
Supplement: Supplementary file 1 — Supplementary information [file 41598_2018_19162_MOESM1_ESM.pdf]

<u>**Supplementary information**</u>

Isoliquiritigenin blunts osteoarthritis by inhibition of bone resorption and angiogenesis in subchondral bone

Baochao Ji [*1] , Zhendong Zhang[*1] , Wentao Guo[1], Hairong Ma[2], Boyong Xu[1], Wenbo Mu[1], Abdusami Amat[1], Li Cao[#1]

Supplementary Figure:

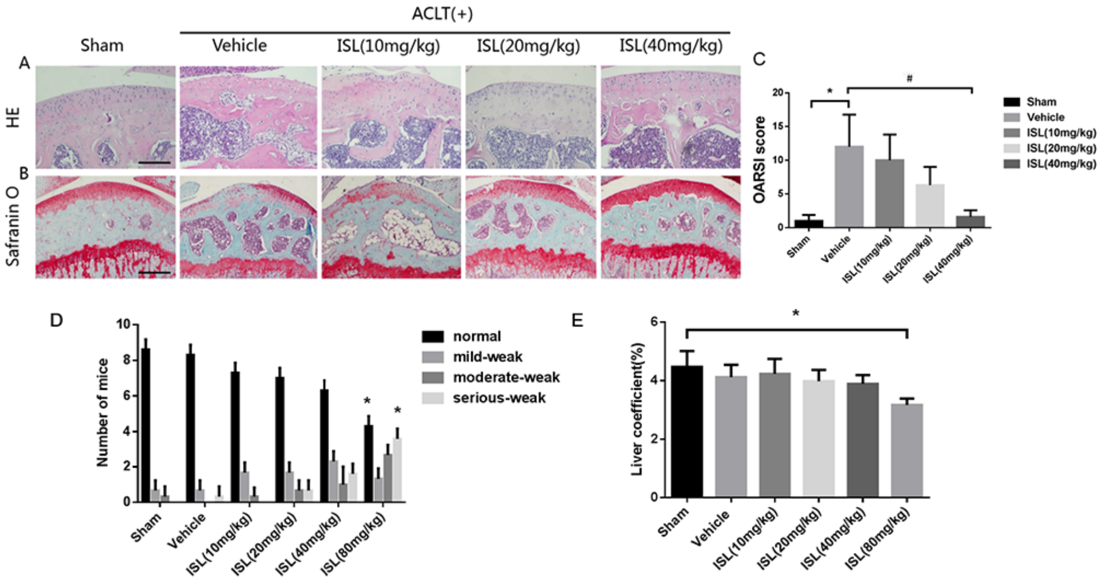

Supplementary Figure 1. The outcome of preliminary experiment. Isoliquiritigenin (ISL) protects articular cartilage after anterior cruciate ligament transection (ACLT) in mice in concentration-dependent manners. (A) The change of thickness of calcified cartilage (CC) and hyaline cartilage (HC) are observed in HE staining (top). Scale bars, 100 mm. (B) Safranin O and fast green staining and Osteoarthritis Research Society International (OARSI)–modified Mankin scores of articular cartilage (C) after surgery indicate proteoglycan loss and cartilage destruction at 60 days post operation. Scale bar, 200 mm. (D) The systemic responses of mice in different concentration groups. (E) The liver coefficient of in different concentration groups. n=10 per group. *p<0.05 compared with sham and #p<0.05 compared with vehicle.

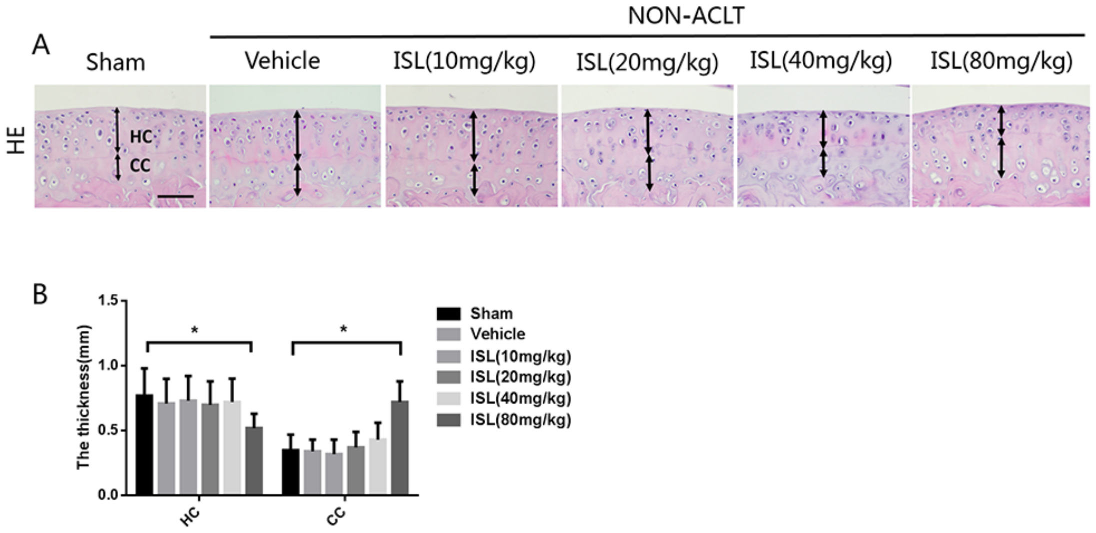

Supplementary Figure 2. The effect of ISL with different concentration on the non-ACLT mice. (A) HE staining and (B) quantitative analysis show the change of thickness of calcified cartilage (CC) and hyaline cartilage (HC) in different concentration group. Scale bars, 100 mm. n=10 per group. *p<0.05 compared with sham.

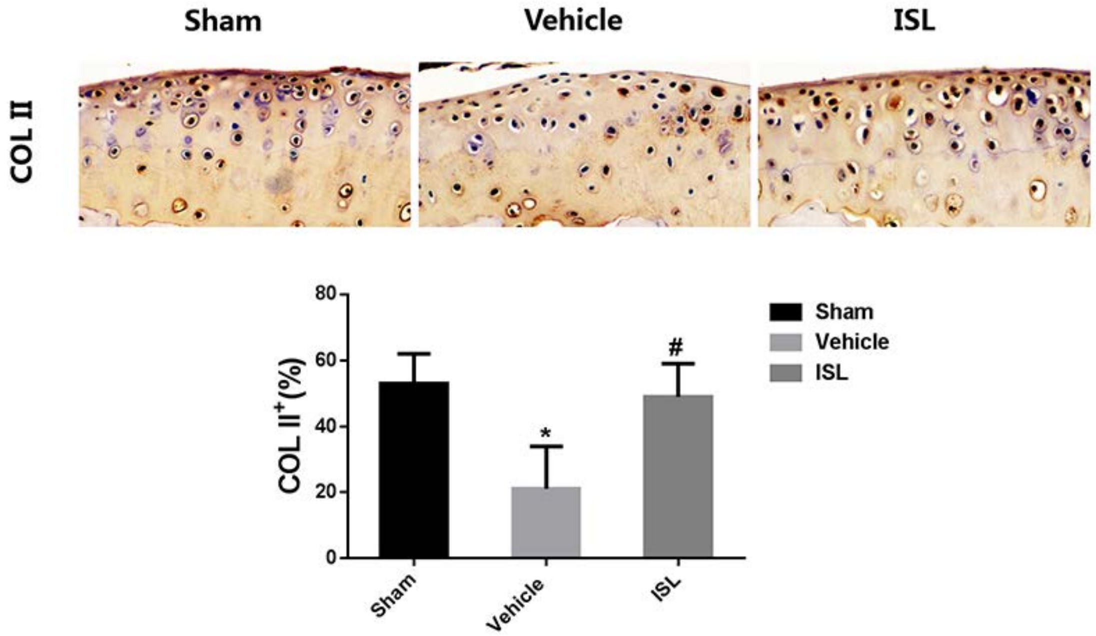

Supplementary Figure 3. The expression of Collagen Ⅱ in articular cartilage 30 days after ACLT were test by immunostaining and quantitative analysis. Scale bar, 100 mm. n=8 per group. *p<0.05 compared with sham or as denoted by bar and #p<0.05 compared with the vehicle.
